# Computational modeling of vacuum-assisted delivery: biomechanics of maternal soft tissues

**DOI:** 10.1007/s10237-025-01977-0

**Published:** 2025-06-19

**Authors:** Rita Moura, Dulce A. Oliveira, Nina Kimmich, Renato M. Natal Jorge, Marco P. L. Parente

**Affiliations:** 1 Faculty of Engineering of the University of Porto, Rua Dr. Roberto Frias s/n, 4200-465 Porto, Portugal; 2 Institute of Science and Innovation in Mechanical and Industrial Engineering, Rua Dr. Roberto Frias 400, 4200-465 Porto, Portugal; 3 Division of Obstetrics, University Hospital of Zurich, Raemistrasse 100, 8091 Zurich, Switzerland

**Keywords:** Assisted delivery simulations, Finite element method, Pelvic floor dysfunctions, Perineal injuries, Vacuum cup

## Abstract

Childbirth is a complex process influenced by physiological, mechanical, and hormonal factors. While natural vaginal delivery is the safest, it is not always feasible due to diverse circumstances. In such cases, assisted delivery techniques, such as vacuum-assisted delivery (VAD), may facilitate vaginal birth. However, this technique can be associated with a higher risk of maternal injuries, potentially resulting in long-term conditions such as pelvic organ prolapse or incontinence. This study investigates the biomechanical impact of VAD on maternal tissues, aiming to reduce these risks. A finite element model was developed to simulate VAD, incorporating maternal musculature, a deformable fetal head, and a vacuum cup. Twelve simulations were conducted, varying contraction durations, resting intervals, and the number of pulls required for fetal extraction. Results revealed that prolonged contraction durations, coupled with extended resting intervals, lead to a reduction in pelvic floor stress. Elevated stress levels were observed when fetal extraction involved two pulls, with an 8.43% decrease in maximum stress from two pulls to four. The peak stress recorded was 0.81 MPa during a 60-second contraction, followed by a 60-second rest period. These findings indicate that longer maneuvers may reduce trauma, as extended pulls allow muscles more time to relax and recover during both contraction and rest phases. Furthermore, an increased number of pulls extends the duration of the maneuver, facilitating fetal rotation and improved adjustment to the birth canal. This study offers crucial insights into the biomechanics of childbirth, providing clinicians with valuable information to enhance maternal outcomes and refine assisted delivery techniques.

## Introduction

Childbirth, though a natural process, is often associated with significant physical trauma for women. The spectrum of childbirth-related injuries ranges from levator ani avulsion to perineal lacerations, all of which can have a profound impact on a woman’s quality of life. Among the techniques used to aid vaginal delivery, vacuum-assisted delivery (VAD) is a common intervention to facilitate the birth process when it is not progressing naturally. VAD rates vary by country, ranging between 10 and 15% in the United States, and 10 to 13% in the United Kingdom (Svelato et al., 2022).

Despite its common occurrence, VAD raises serious concerns regarding maternal pelvic floor dysfunction. Substantial evidence indicates that instrumental deliveries significantly increase maternal morbidity, including perineal trauma and long-term urinary and fecal incontinence (Ali and Norwitz, 2009). Notably, studies indicate that around 30% of women who underwent VAD reported new-onset incontinence of any kind (Sng et al., 2022), and that maternal soft tissue trauma can range between 21 and 36% (Ali and Norwitz, 2009), underscoring the clinical significance of these complications. Of particular concern is the technique’s association with levator ani muscle (LAM) injuries, with avulsion rates reported as high as 34% in vacuum extractions (García Mejido et al., 2017). Additionally, VAD is linked to a higher rate of third- and fourth-degree perineal lacerations (10%) compared to spontaneous vaginal deliveries (2%) (Angioli et al., 2000). Although forceps deliveries are associated with an even higher incidence of maternal complications, their use has declined substantially in recent decades. As VAD becomes the predominant operative vaginal delivery technique, there is a growing need for further investigation into its short- and long-term maternal outcomes.

From a clinical perspective, there are still some unanswered questions. Obstetricians are uncertain about how long vacuum extraction should last, how long the vacuum cup should be in place, and the biomechanical effects of its duration on the pelvic floor and perineum. Additionally, the acceptable number of pulls is also unknown, and most guidelines are based on observational studies. The relationship between the number of pulls and adverse outcomes remains unclear (Kamijo et al., 2021). Given the significant impact of assisted vaginal birth on maternal health and the lack of conclusive evidence on the specific risks associated with vacuum extraction, there is an urgent need for extensive studies to elucidate the biomechanical factors underlying childbirth-related injuries.

Computational models offer significant value in the quantitative analysis of the physiology of labor. Childbirth simulations can overcome the limitations of traditional physical models and invasive procedures, providing a deeper understanding of the mechanisms involved in vaginal delivery and the resulting maternal and fetal injuries (Hoyte and Damaser, 2016). Several computational models have been developed to simulate normal vaginal delivery. Previous research has explored mechanical changes in the pelvic floor muscles (PFM) during delivery, quantified muscle damage, assessed the trajectory of the fetus, studied fetal head molding, examined the effects of various obstetric procedures, and conducted time-dependent analyses (Parente et al., 2008; Buttin et al., 2013; Oliveira et al., 2016a; Oliveira et al., 2016b; Vila Pouca et al., 2017; Moura et al., 2020). Additionally, models of the pelvic cavity have been used to evaluate the shape of the fetal head and investigate urogenital prolapse (Lepage et al., 2015; Yan et al., 2015). More recently, childbirth computational models have incorporated perineal tissues in the analysis of maternal injuries to improve the ability to predict distinct lesions (Cechova et al., 2021; Moura et al., 2024b).

Although there are numerous studies in the literature on VAD, finite element simulations of this delivery method have not been properly addressed. Only a handful of studies have focused on simulating assisted delivery. For example, Lapeer et al. (2014) used a finite element analysis to simulate vacuum extraction during delivery, while Huang et al. (2022) examined the effects of different sizes of vacuum extractors during delivery. In the area of forceps delivery, Su et al. (2016) investigated the influence of forceps blade angles on the fetal head. However, these studies primarily focused on the deformations or stresses on the fetal head induced by these procedures and neglected to evaluate their effects on maternal tissues.

The primary objective of this study is to investigate the effects of VAD on maternal tissues, with particular emphasis on the PFM and perineum. A key focus is to identify the regions most susceptible to injury, offering a clearer understanding of the biomechanical impact of VAD on these critical structures. The study also explores how variations in the duration of contractions and resting periods between pulls influence maternal tissue outcomes, along with the correlation between the number of pulls required for successful delivery and the degree of tissue trauma. By employing a computational approach, this work aims to provide insights that could inform clinical practice and improve maternal health outcomes.

## Methodology

### Biomechanical model

**Fig. 1:**
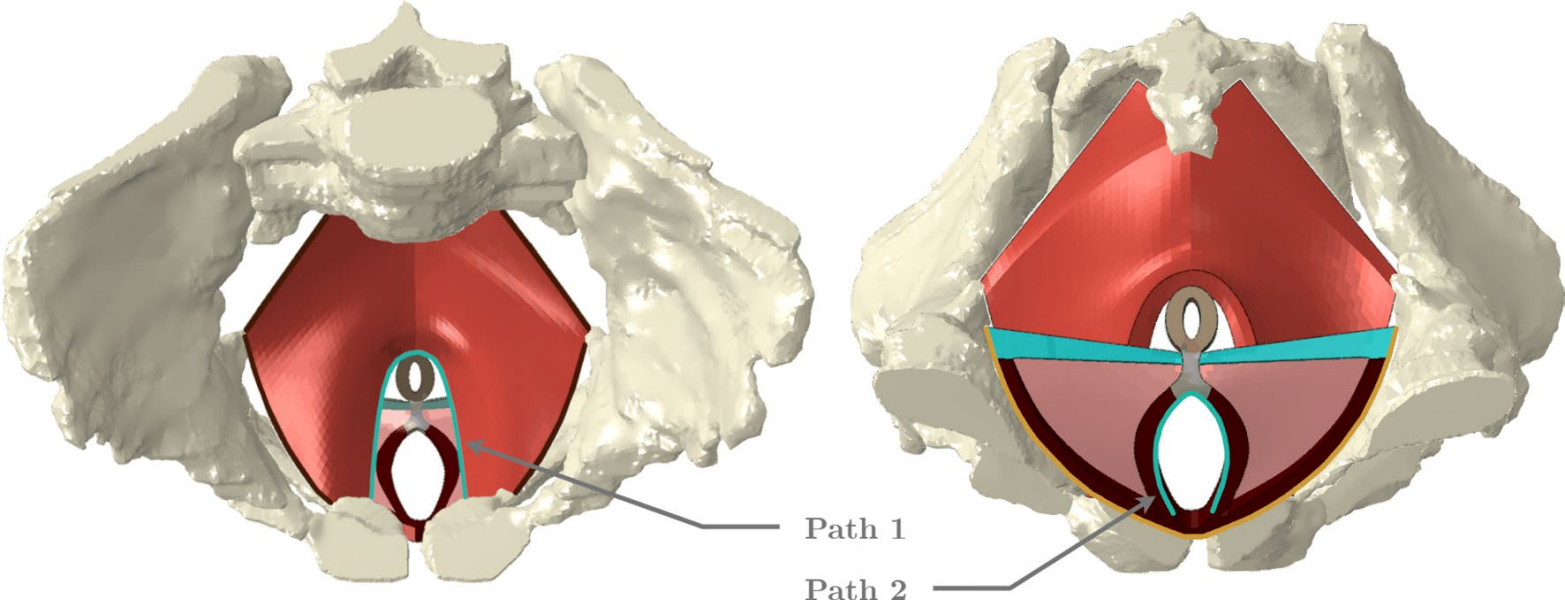
Geometric model of the PFM, in red, and perineum. The perineal body is presented in gray, the external anal sphincter in brown, the ischiocavernosus and bulbospongiosus muscles in dark red, the superficial transverse perineal muscle in blue, and deep transverse perineal muscles in pink. The blue lines correspond to two paths defined on the levator hiatus, path 1, and the urogenital hiatus, path 2 (the space through which the urethra and the vagina pass). The dark brown line corresponds to the fixed nodes of the PFM, while the yellow line corresponds to the constrained nodes of the ischiocavernosus and superficial transverse perineal muscles. The left figure corresponds to a superior view of the female pelvis and the right figure to an inferior view

A finite element model of the PFM, perineal structures, and fetal head was used to simulate VAD. The model of the PFM and perineum was developed in a previous study by Moura et al. (2024b), including the levator ani and coccygeus muscles, perineal body, ischiocavernosus, bulbospongiosus, superficial and deep transverse perineal muscles, and the superficial layer of the external anal sphincter. The PFM mesh comprises 5712 nodes and 3618 hexahedral elements (C3D8H), while the perineum has 7253 nodes and 4862 C3D8H elements. Hybrid elements were used in this study because the modeled tissues are hyperelastic and nearly incompressible. In such cases, standard elements can suffer from volumetric locking. The hybrid formulation introduces an additional degree of freedom to enforce incompressibility through a pressure variable, which significantly enhances numerical stability. The complete finite element model used is presented in Fig. 1, and further details can be found in the aforementioned work.

The fetal head was modeled with dimensions consistent with the literature for a full-term fetus (Parente et al., 2008). It was represented as a deformable structure consisting of 4388 nodes and 19537 tetrahedral elements (C3D4H). To manage the complexity of the numerical simulations, the head was modeled as a single unified structure rather than being divided into separate components. This approach allows the head to undergo deformation while minimizing contact conditions and interactions that could hinder the convergence of the numerical simulations.

**Fig. 2:**
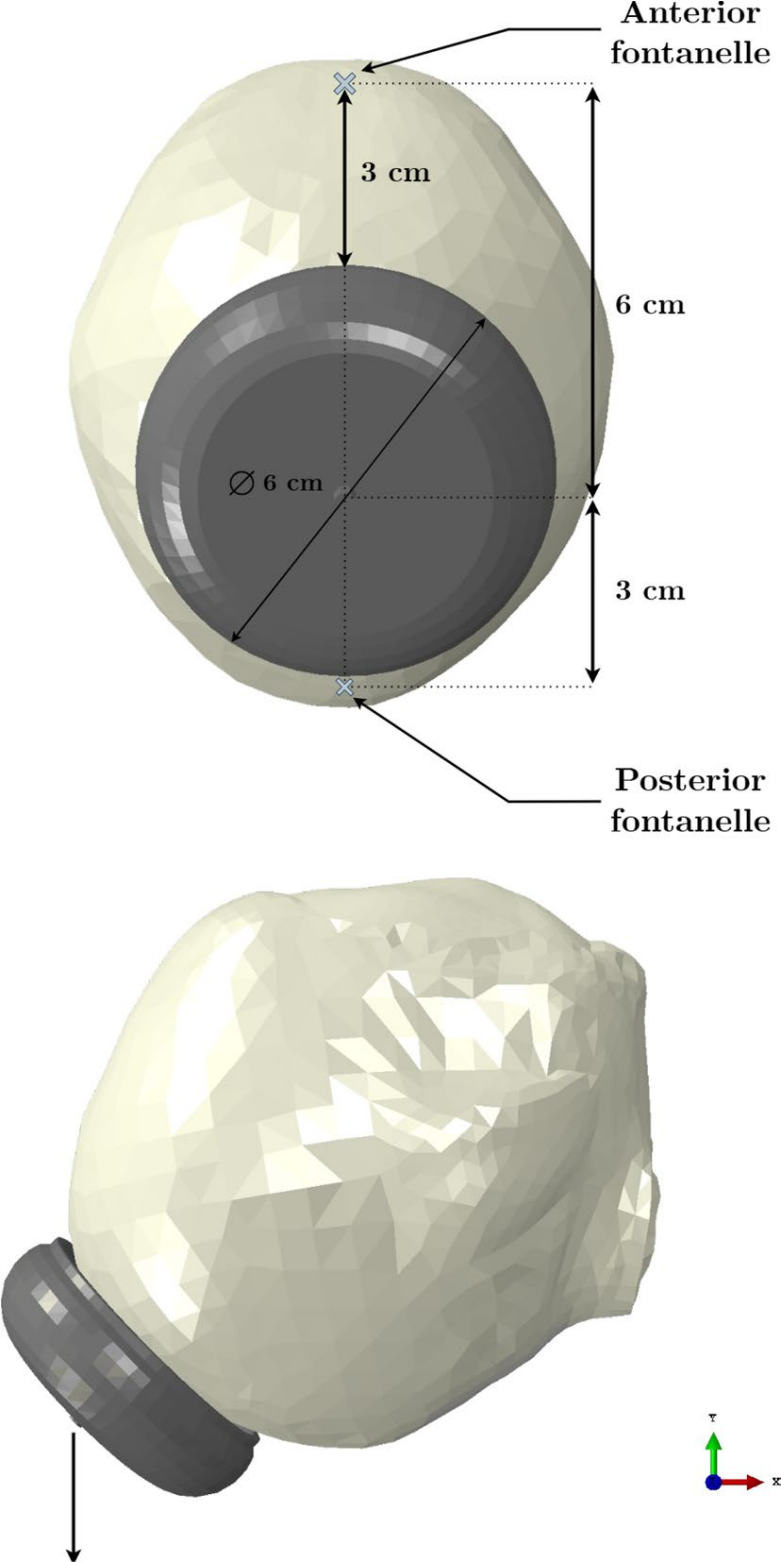
Representation of the finite element model of the fetal head and vacuum cup. The vacuum cup is positioned at the flexion point of the fetal head, showing the distances to the anterior and posterior fontanelles. The black arrow at the tip of the vacuum cup indicates the direction of the imposed displacement

Lastly, the finite element model of the vacuum cup corresponds to the Kiwi Omnicup®Complete Vacuum Delivery System (Laborie Medical Technologies, New Hampshire, USA). A diameter of 60 mm and a height of 20 mm were the defined measurements. The mesh is composed of 7146 of type C3D4 and 13553 nodes. The vacuum cup was modeled as a rigid structure. Figure 2 shows the finite element model of the fetal head and vacuum cup, with the vacuum cup positioned on the flexion point of the fetal head. This point is located on the sagittal suture, 6 cm behind the anterior fontanelle and 3 cm anterior to the posterior fontanelle. The center of the cup is aligned with the flexion point, and with a diameter of 60 mm, its brim reaches the posterior fontanelle.

### Numerical simulations

The finite element simulations were performed in Abaqus/Standard®software under a static analysis to mimic a VAD with the fetus in the vertex presentation and occipito-anterior position.

#### Boundary conditions and interactions

The following boundary conditions were applied to the simulations: the nodes at the extremities of the PFM and the superficial transverse perineal muscles, as well as those of the bulbospongiosus muscles near the pubic symphysis, were fixed. The external nodes of the ischiocavernosus muscles, which attach to the ischial bone, were fixed along the vertical direction to reflect this attachment. The boundary conditions are represented in Fig. 1.

During the simulations, interactions between the PFM, perineum, fetal head and vacuum cup were established using the standard Abaqus®contact algorithm. The Augmented Lagrange contact method was used to define the contact between these structures and to facilitate surface interactions. A tie constraint was established between the fetal head and the vacuum cup to ensure they remain connected throughout the simulation.

#### Vacuum-assisted delivery dynamics

In a VAD, the vacuum cup is typically introduced when the fetal head is at a station between +1 and +3, indicating an advanced but stalled stage of labor. This scenario corresponds to the early stages of the finite element simulations usually performed to replicate childbirth. In the present study, the simulation begins with the engagement of the fetal head into the PFM. This process has a duration of three hours to mimic the conditions of prolonged labor, where vacuum extraction is often indicated. After this phase, the assisted delivery simulation starts. As the fetal head is not visible from the outside, the vacuum cup must be inserted through the perineum. The initial engagement of the fetal head on the PFM is crucial, as the muscles already experience some degree of deformation when the vacuum cup is introduced, facilitating its proper placement. The next step involves positioning the vacuum cup alongside the perineal region into the vagina and through the PFM on the fetal head, enabling control over its descent.

**Fig. 3:**
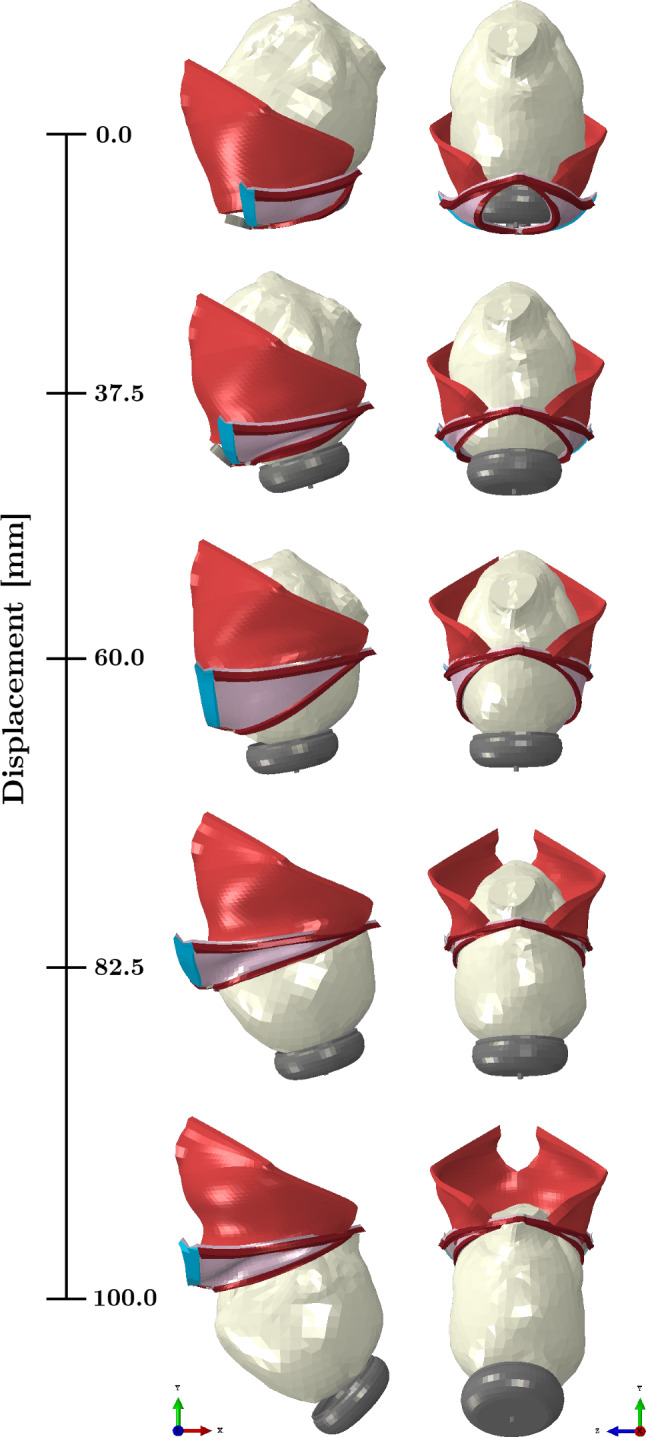
Dynamics of the VAD simulation. Lateral and anterior views of the fetal head descent corresponding to the vertical displacement of the vacuum cup from 0 to 100 mm

The movements of the vacuum cup are controlled by a reference node, which defines its descent and rotation. Consequently, the initial phase of the simulation focuses on “delivering" the vacuum cup by maneuvering it through the PFM and the urogenital hiatus. Once this stage is completed, the simulation advances to the extraction of the fetal head. The different stages of the VAD simulation can be seen in Fig. 3.

A total of twelve simulations of VAD were performed, each designed to replicate realistic clinical scenarios. Key parameters were varied, including the contraction duration, during which the obstetrician pulls the fetal head, the resting period between contractions, and the total number of pulls required to extract the fetus. Since the uterus is not included in the finite element model, uterine contractions are represented indirectly by prescribing specific fetal descent dynamics, applying a downward displacement during the pulling phase and maintaining the head position during the resting phase. Based on cardiotocographic data and input from experienced obstetricians, the duration of uterine contractions typically ranges from 60 to 90 s, with rest intervals between contractions varying from 60 to 180 s. Using this information, extreme values were selected for the simulations performed: contraction durations of 60 and 90 s, and resting intervals of 60 and 180 s. Furthermore, scenarios involving two, three, and four pulls were tested. The combination of these parameters resulted in twelve distinct simulation setups.

#### Calculations

The maximum principal stress was analyzed along two predefined paths in the PFM: one in the inferior portion corresponding to the levator hiatus, and another along the urogenital hiatus, as illustrated in Fig. 1. Stress values were recorded throughout the vertical displacement of the vacuum cup. Stretch was also evaluated along these paths, defined as the ratio between the current length and the original length. The forces applied to the vacuum cup during fetal extraction were measured, considering only the force component in the y-direction, as the head is pulled along this axis.

Maximum principal stress and forces were chosen as the primary variables for comparing results across the different cases. Since the geometry of the muscles and fetal head remains unchanged in all simulations, the resulting stretch and deformation of the maternal muscles are expected to remain constant.

### Mechanical characterization

The PFM and most of the perineal muscles, namely the ischiocavernosus, superficial and deep transverse perineal muscles, were characterized by the Martins transversely isotropic hyperelastic constitutive model (Martins et al., 1998). The strain energy density function is given by Equation 1.

1 $$\begin{aligned} U = U_m + U_f + U_J \end{aligned}$$

where $U_m$ corresponds to the isotropic strain energy stored in the isotropic matrix (Equation 2), $U_{f}$ represent the strain energy stored in each muscle fiber (Equation 3), and $U_J$ is related to the volume change (Equation 4).

2 $$\begin{aligned} U_m = c \Bigl \{ e^{b \big (\overline{I}_1^C - 3\big )} - 1 \Bigl \} \end{aligned}$$

where $\overline{I}_1^C$ is the first invariant of the right Cauchy–Green strain tensor with the volume change eliminated, and *c* and *b* are constitutive parameters related to the isotropic part.

3 $$\begin{aligned} \begin{aligned} U_f&= \underbrace{A \Bigl \{ e^{\bigr [a \bigl (\overline{\lambda }_f - 1 \bigl )^2 \bigr ]} - 1 \Bigl \} }_{{U_{f_{PE}}}} \\&\quad + \underbrace{\theta ~T_0^{M} \int _{1}^{\overline{\lambda }_f} \Bigr [- 4 \bigl (\overline{\lambda }_f - 1 \bigl )^2 + 1 \Bigr ] ~ d\overline{\lambda }_f}_{{U_{f_{SE}}}} \end{aligned} \end{aligned}$$

where $U_{f_{PE}}$ and $U_{f_{SE}}$ represent, respectively, the passive and active parts of the strain energy stored in each muscle fiber, $\overline{\lambda }_f$ represents the stretch ratio of the muscle fibers, $T_0^{M}$ is the maximum muscle tension produced by the muscle at resting, $\theta \in [0,1]$ defines the level of activation, and *A* and *a* are constitutive parameters related to the muscle fibers.

4 $$\begin{aligned} U_J = \frac{1}{D} (J - 1)^2 \end{aligned}$$

$J = det({\textbf {F}})$ is the volume ratio, ${\textbf {F}}$ is the deformation gradient, and *D* is a constitutive parameter.

In this work, only the passive behavior of the muscles was included. While muscles retain some residual tone even under anesthesia, it was assumed $\theta = 0$ for all the simulations, as a reasonable approximation for a relaxed state. This condition is observed in more than 60% of women who have vaginal birth, according to the Centers for Disease Control (Osterman and Martin, 2011).

The viscoelastic contribution is represented by the generalized Maxwell model (Vila Pouca et al., 2017). This model was chosen since it has been successfully used in previous studies to represent the viscous behavior of soft biological tissues, particularly the PFM. Its mathematical simplicity, characterized by a minimal number of parameters, enables efficient and accurate fitting to the available experimental data. The implementation includes the addition of a dissipative potential to the strain energy function (Equation 5), describing the non-equilibrium state.

5 $$\begin{aligned} U_{vsc} = \sum _{\alpha = 1}^{m} \gamma _\alpha (\overline{{\textbf {C}}}, \varvec{\Gamma }_\alpha ) \end{aligned}$$

where $\gamma _\alpha$ represents the dissipative potential, which is a function of the modified right Cauchy–Green strain tensor, $\overline{{\textbf {C}}}$, and a set of strain-like internal variables denoted by $\varvec{\Gamma }_\alpha$. The latter characterizes the relaxation and/or creep behavior of the material. The viscoelastic behavior is modeled by $\alpha$=1,...,m viscoelastic processes.

The perineal body and bulbospongiosus muscle were considered linear elastic (E = 23.8 kPa and $\nu$ = 0.49) (Zhou et al., 2023). The Neo-Hookean model was used to characterize the external anal sphincter, with a material constant defined as $c = 0.1$ MPa, as explained in Moura et al. (2024b). The material parameters used to characterize the PFM and the perineal muscles are presented in Table 1.

**Table 1:** Material parameters used to characterize the pelvic floor and perineal

Hyperelastic parameters (Parente et al., 2009)	Viscoelastic parameters (Vila Pouca et al., 2017)			
c = 0.0185 MPa	$B_{m1}$ = 1.5 (–)	$\tau _{m1}$ = 20 s	$B_{f1}$ = 1.2 (–)	$\tau _{f1}$ = 0.9 s
b = 1.1730 (–)	$B_{m2}$ = 0.7 (–)	$\tau _{m2}$ = 400 s	$B_{f2}$ = 0.5 (–)	$\tau _{f2}$ = 250 s
A = 0.0280 MPa	$B_{m3}$ = 0.5 (–)	$\tau _{m3}$ = 5000 s	$B_{f3}$ = 0.3 (–)	$\tau _{f3}$ = 3500 s
a = 0.6215 (–)				

**Fig. 4:**
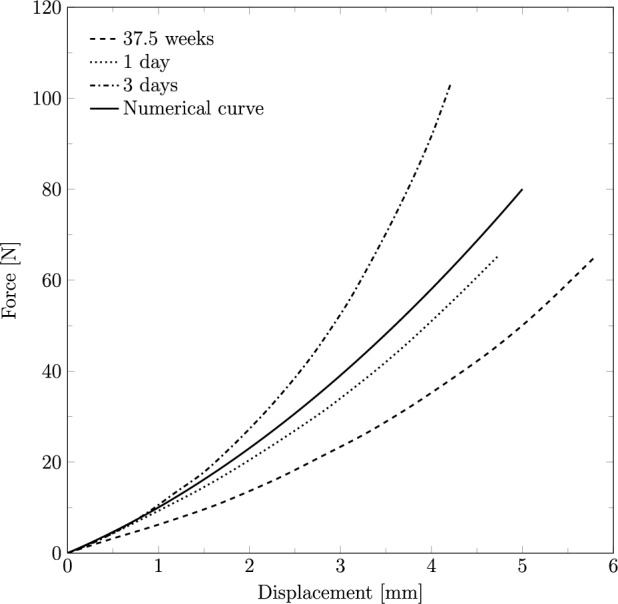
Force–deflection curve obtained from the antero-posterior compression test for the fetal head finite element model, compared to the experimental curves by Loyd (2011)

As previously mentioned, the fetal head was considered a deformable structure. The head was characterized using a linear elastic constitutive model, with a Young’s modulus of 1.1 MPa and a Poisson’s ratio of 0.25. To calibrate the parameters used in this characterization, an antero-posterior compression test performed by Loyd (2011) was replicated. The Young’s modulus was optimized until the numerical curve matched the experimental curves, which were obtained from neonates aged 37.5 weeks, 1 day, and 3 days. The setup of the compression simulations was replicated according to the experimental tests. The head was placed between two rigid plates, and a displacement of 5.0 mm was applied in the antero-posterior direction. The resulting force–deflection curve is presented in Fig. 4, alongside data from the literature for comparison.

## Vacuum-assisted delivery simulations

### Analysis of the maternal structures

**Fig. 5:**
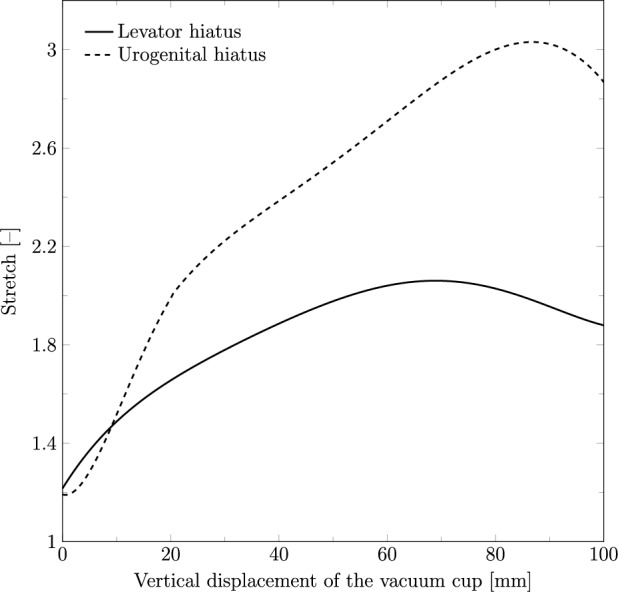
Stretch of the levator hiatus (PFM) and urogenital hiatus (perineum) during the vertical displacement of the vacuum cup

Figure 5 illustrates the stretch measured on the levator hiatus and urogenital hiatus during the vertical displacement of the vacuum cup. The maximum stretch of the levator hiatus reached 2.09 at a descent of 69.91 mm, while the perineum exhibited a stretch of 3.01 at a descent of 79.51 mm.

**Fig. 6:**
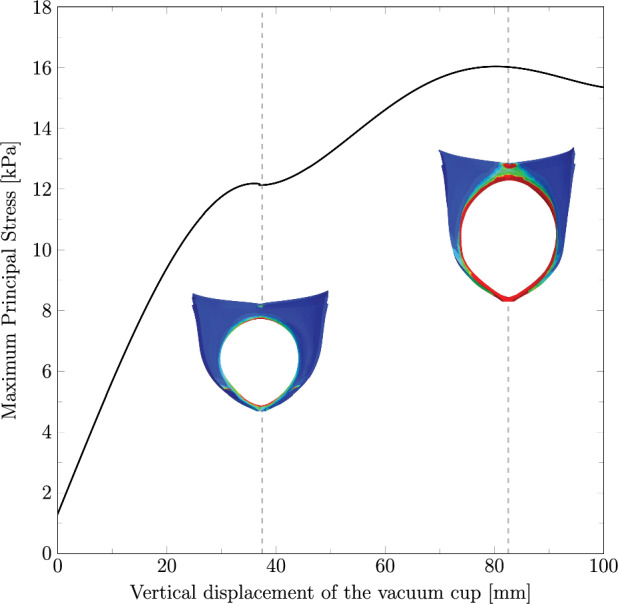
Maximum principal stress (in MPa) along the vertical displacement on the vacuum cup measured on Path 2, corresponding to the urogenital hiatus. The results include the finite element model of the perineum for vertical displacements of 37.5 mm and 82.5 mm, highlighting the stress distribution and identifying the most critical regions. Before reaching 37.5 mm, the stretch is primarily due to the extraction of the vacuum cup

The maximum principal stress on the urogenital hiatus was also evaluated. As the bulbospongiosus muscle and perineal body were modeled without viscoelastic properties in this study, the stress distribution in these structures remains unchanged across the different cases under analysis. Consequently, Fig. 6 shows the stress results on the urogenital hiatus obtained for all cases during the vertical displacement of the vacuum cup.

Based on the analysis of the maternal structures, the following results will focus on the vertical displacement of the vacuum cup between 55 and 80 mm, as this range represents the most critical interval for the pelvic floor.

### Effect of the contraction duration and resting stages

**Fig. 7:**
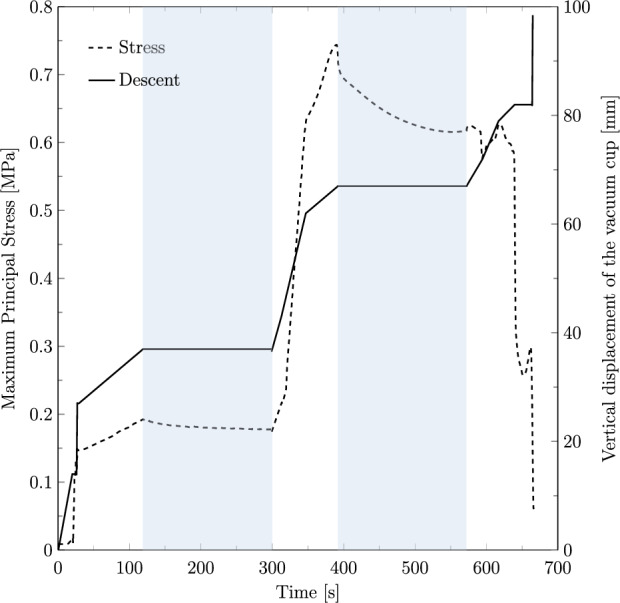
Time evolution of the maximum principal stress in the fetal head (left axis) and the vertical displacement of the vacuum cup (right axis) during the simulated VAD. The shaded blue areas indicate resting phases, while the white areas represent pulling phases

Figure 7 shows the time evolution of the maximum principal stress at the levator hiatus and the vertical displacement of the vacuum cup. The plot represents a VAD simulation with three pulls, 90 s of contraction and 180 s of rest. The results reveal a notable reduction in stress during the resting phases, particularly in the second rest period with a stress relaxation of 19.22%, underscoring the muscle recovery effect facilitated by the intervals between contractions.

**Fig. 8:**
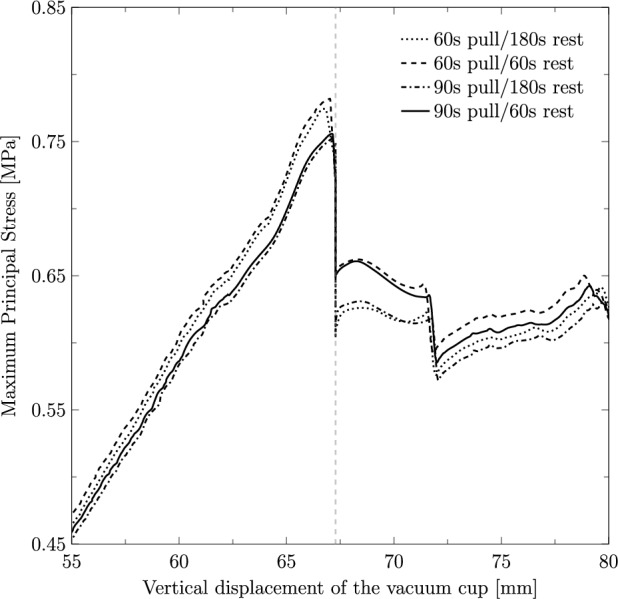
Maximum principal stress (in MPa) along the vertical displacement of the vacuum cup measured on the PFM path for the different cases under study. The vertical dashed line indicates the displacement at which the resting stage is defined

To investigate the effect of contraction duration and resting periods on the PFM, the maximum principal stress on the levator hiatus was measured along the vertical displacement of the vacuum cup, as shown in Fig. 8. The results demonstrate that shorter contraction and resting times result in higher stress values, whereas longer contraction and resting periods lead to lower stress levels. The highest recorded stress was 0.78 MPa, observed in the simulation with 60 s of contraction and 60 s of rest, corresponding to a fetal descent of 67.03 mm.

The force applied to the vacuum cup to extract the fetus was measured in the y-direction, corresponding to the vertical displacement of the vacuum cup from the moment it is attached to the fetal head. The maximum recorded force was 130.10 N at a vertical displacement of 63.69 mm, during a contraction phase lasting 60 s, followed by a resting phase of equal duration. The descent moment can be associated with the moment of maximum stretch of the levator hiatus. The results show a similar trend to the maximum principal stresses observed across the different simulated cases.

### Impact of the number of pulls

**Fig. 9:**
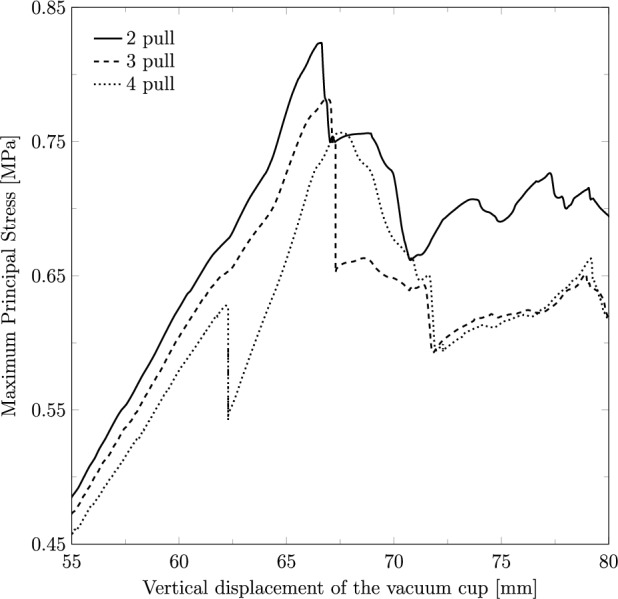
Maximum principal stress (in MPa) along the vertical displacement on the vacuum cup considering the fetal extraction with two, three and four pulls

As the highest stress values were recorded in the 60 s contraction and 60 s rest scenario, the impact of the number of pulls was analyzed for these specific contraction and rest durations. Figure 9 illustrates the maximum principal stress observed during the VAD simulations, comparing fetal extraction scenarios with two, three, and four pulls. The results indicate that using only two pulls leads to higher stress on the muscles, with a maximum stress of 0.81 MPa for a vertical displacement of 66.28 mm, compared to three pulls, which result in a maximum stress of 0.78 MPa for a displacement of 66.82 mm, and four pulls, which lead to a maximum stress of 0.76 MPa for a displacement of 67.54 mm.

**Fig. 10:**
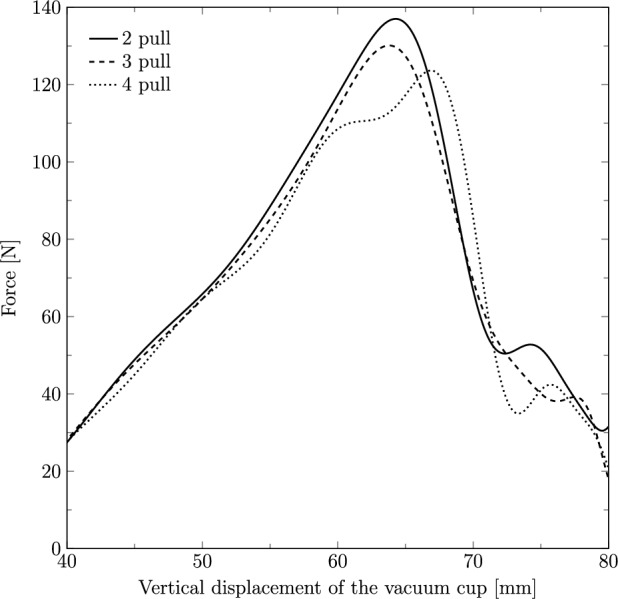
Force (in N) applied on the vacuum cup to extract the fetal head along its vertical displacement through the birth canal with two, three and four pulls

The force applied to the vacuum cup to extract the fetus was measured in the y-direction along its vertical displacement for two, three, and four pulls (Fig. 10). The maximum forces recorded were 136.99 N, 130.10 N and 123.63 N, corresponding to vertical displacements of 64.24 mm, 63.69 mm, and 66.98 mm, respectively.

## Discussion

Assisted delivery is a widely used obstetric technique designed to facilitate labor progression when complications arise that hinder natural delivery or when the birth needs to be accelerated due to imminent fetal compromise. Among these techniques, vacuum-assisted birth is commonly used, although it is associated with high rates of pelvic floor trauma and perineal lesions. Despite its widespread use, the biomechanical effects of VAD on maternal tissues remain poorly understood. This study seeks to address this knowledge gap by examining the mechanical impact of this technique on maternal tissues. The novelty of this work lies in the biomechanical analysis of VAD, with a focus on specific controllable factors that may play a critical role in the prevention of pelvic floor dysfunction.

When analyzing the maternal structures, the levator hiatus was found to stretch to approximately twice its initial length, consistent with the findings of Moura et al. (2024b), where vaginal delivery was simulated using the same finite element model of the PFM and perineum. Similarly, the urogenital hiatus stretched approximately three times its initial length in both studies. These results suggest that VAD does not significantly impact muscle stretch, as this is primarily a geometrical feature influenced by fetal head size and the intrinsic dimensions of the muscles. However, in the VAD simulations, the maximum stretch occurred at an earlier stage, indicating that the use of the cup might induce faster and earlier muscle opening compared to normal vaginal delivery. This earlier stretch might have clinical implications, potentially increasing the risk of muscle injury or contributing to long-term pelvic floor dysfunction, as reported in previous clinical studies.

For the analysis of the maternal structures, it is crucial to identify the regions subjected to higher stress levels, as these are more prone to injury during VAD. The perineum representation in Fig. 6 highlights that during maximum perineal stretch, the most affected regions are the anterior area near the attachment to the pubic symphysis and the middle posterior region connecting to the perineal body. Additionally, the region of the perineal body adjacent to the anal sphincter also experiences significant stress, indicating a potential risk for obstetric anal sphincter injuries (OASIS). These findings are consistent with the literature, which commonly reports perineal tears in the posterior region and OASIS at the connection with the anal sphincter. Regarding the PFM, the highest stress concentrations are observed in the central portion of the muscle and at the attachment points to the surrounding structures, as reported in previous numerical studies.

Distinct contraction and resting stages were simulated to evaluate their impact on the maternal muscles. The results show that the highest muscle stresses were observed for a contraction duration of 60 s combined with a resting stage of 60 s. This scenario induces higher stresses as the shorter resting time limits the muscles’ ability to recover and adapt, resulting in a more abrupt descent. This effect can be attributed to the viscoelastic properties of pelvic floor tissues, which require adequate time to recover following deformation. Interestingly, Fig. 8 shows that the stress levels for the 60 s contraction−180 s rest case are nearly identical to those of the 60 s contraction−60 s rest case during the contraction phase. However, after the resting stage, the stresses significantly decrease, with a reduction of approximately 21.71%, and approach the values observed in the 90 s contraction−180 s rest case. This suggests that the duration of the resting stage has a greater influence on muscle stress recovery than the contraction duration itself. Clinically, this highlights the importance of optimizing contraction-to-resting time ratios during labor management to protect the maternal muscles. Chill et al. (2024) concluded that a shorter duration of VAD can increase the risk of OASIS since an increased procedure time may afford perineal tissues the opportunity to stretch and accommodate to the fetal head. This is in line with our findings that longer deliveries might be beneficial to the PFM, as the stresses are lower.

Another objective of this study was to determine whether the number of times the obstetrician must pull to deliver the fetus affects the maternal muscles. This analysis focused on the PFM, and the results revealed that the number of pulls significantly influences muscle stress. Specifically, the stresses on the muscles were found to be higher when the fetus was extracted with only two pulls compared to four pulls, with an 8.43% difference in the maximum stress values between these two scenarios. This indicates that fewer pulls result in more intense muscle effort, which may increase the risk of injury. These findings are consistent with the results observed for the variations in the contraction and resting stages. When the fetal head is extracted using a higher number of pulls, there is more time for the muscles to relax between pulls, allowing for better adjustment and leading to lower stress.

Additionally, the force applied to the vacuum cup was analyzed across different number of pulls. The results showed that a higher force was required for extraction with two pulls compared to the other cases. The increased force required in these scenarios could potentially lead to a higher risk of maternal trauma, such as perineal tears or pelvic floor damage, as well as a higher risk of cup detachment from the fetal head, which may increase the risk of intracranial bleeding. Therefore, obstetricians may benefit from considering a more gradual approach to assisted delivery, with careful attention to the timing and number of pulls applied. Literature indicates that a traction force of 115 N to 125 N is sufficient for successful delivery and that this traction should not exceed 135 N (Goordyal et al., 2021). The values obtained in the present study fall within this range, thereby supporting the validity of the finite element model and the approach implemented.

The findings of this study provide valuable insights for obstetric practice regarding the biomechanical implications of VAD. From a mechanical perspective, with a focus on maternal health, the study concludes that longer contraction durations and extended rest periods are beneficial for maternal muscles. These intervals allow muscles more time to adapt and stretch, thereby reducing the risk of potential injuries caused by abrupt deformations. Since these factors can sometimes be influenced by medication, in situations where there is no immediate risk to the fetus or the mother, these recommendations may help achieve the best possible outcomes in what is already a challenging and potentially traumatic scenario. Additionally, the finding that a higher number of pulls can benefit maternal muscles offers a practical clinical guideline. Obstetricians could aim to avoid excessive force during individual pulls and instead utilize more contractions, optimizing relaxation time for the muscles and minimizing excessive deformation.

However, it is important to balance these benefits against potential risks to the fetus, such as hypoxia, which may arise from prolonged delivery times. Future research should explore this balance in greater detail, with a focus on developing clinical guidelines that prioritize both maternal and fetal well-being. To establish clinical guidelines, a structured approach is required, beginning with the integration of real-world clinical data, followed by model calibration and validation against observed outcomes such as perineal lacerations and pelvic floor injuries. Once validated, the model can be used to simulate a range of clinical scenarios, assess the impact of varying delivery forces, define risk thresholds, and guide safer obstetric practices.

Although the tridimensional model presented in this work has specific dimensions corresponding to an average of the literature, both the PFM, the perineal structures and the fetal head can be modified to adapt to the geometric features of a specific woman with the mesh morphing algorithm implemented in Moura et al. (2024a). After gathering the relevant dimensions on pre-birth ultrasounds, the simulations can be performed patient specifically and provide insights to the clinicians. Additionally, when combined with patient-specific data, these models can be used to define quantitative criteria for proposing optimal delivery conditions and assessing the risk of potential injuries. With proper validation against clinical data, quantitative thresholds could be established, for example, the maximum extraction force beyond which the risk of injury significantly increases, the maximum muscle stretch that can occur without causing injury, and the stress values beyond which the likelihood of postpartum pelvic floor disorders or perineal lacerations becomes significant. To reduce model uncertainties, capturing the dynamics of labor using MRI, as demonstrated by Ami et al. (2016), could be a valuable approach, along with obtaining *in vivo* mechanical properties using recent techniques such as shear wave elastography.

This approach could serve as a decision support tool, enabling obstetricians to evaluate the potential mechanical outcomes of different delivery strategies in a personalized manner. By identifying patients at higher risk of pelvic floor trauma, tailored labor management protocols could be implemented to mitigate these risks. Additionally, patient-specific models could assist in optimizing the use of pharmaceutical agents or physical therapy as preventive measures in the prenatal period. Future developments in this field, particularly when combined with machine learning algorithms, could further enhance the predictive capabilities of such models, ultimately contributing to more individualized and safer obstetric care.

The proposed work presents several limitations. Firstly, although the fetal head is modeled as a deformable structure, it is represented as a single entity without distinguishing between cranial bones, sutures, fontanelles, or brain tissue. Incorporating these anatomical components could potentially allow for greater deformation of the fetal head, thereby reducing maternal muscle stretch. However, this would significantly increase the complexity and computational cost of the simulations. The model also omits the fetal body, which would likely not affect the results of the simulations performed but could be important for simulating other maneuvers or delivery strategies in the context of VAD. Another limitation lies in the vacuum cup model used for the simulations, as only one specific type was considered despite the existence of multiple designs, sizes, and materials. Assessing different cup models could help determine their biomechanical impact. Additionally, the placement of the vacuum cup depends on both the head’s size and shape, yet only one fetal head model with fixed dimensions was tested. Simulations with varying head geometries would provide a broader understanding of this effect. The interaction between the fetal head and the vacuum cup is also simplified, as the suction mechanism of the cup is not modeled. Furthermore, the finite element model does not account for additional surrounding structures and fluids, which could influence the delivery process. Lastly, another limitation of this study is the absence of a dedicated mesh sensitivity analysis within the current simulation setup. Although the muscle model used has been previously validated for childbirth biomechanics, mesh density can affect localized stress values. Consequently, finer discretization may reveal additional detail in regions of high strain.

## Conclusion

This study presents a novel approach to analyzing the impact of assisted delivery techniques on maternal tissues. By addressing critical, previously unanswered clinical questions, this approach could shift clinicians’ perspectives and guide the development of new guidelines that integrate biomechanical insights. The findings offer valuable knowledge on the biomechanics of assisted delivery, emphasizing the importance of optimizing techniques to minimize maternal trauma. These insights have the potential to influence clinical guidelines and future research directions, ultimately contributing to improved maternal health outcomes. Future research should expand to include other relevant structures, such as the uterus and cervix, and provide a more detailed characterization of the fetus, allowing for a comprehensive analysis of the process as it affects fetal tissues as well. Moreover, simulating the fetus in various positions, particularly the occipito-posterior position, would provide valuable insights into positional effects and enhance the ability to model a wider range of clinical scenarios. Nonetheless, this study represents an important step forward in advancing the current understanding of childbirth biomechanics and in developing tools that could enable clinicians to make more precise, case-specific decisions for improved outcomes.

## Data Availability

No datasets were generated or analyzed during the current study.
